# Implementation of a Tool to Modify Behavior in a Chronic Disease Management Program

**DOI:** 10.4061/2011/215842

**Published:** 2011-03-08

**Authors:** Nicole D. Gillespie, Thomas L. Lenz

**Affiliations:** ^1^Community Pharmacy Practice Residency Program, Creighton University, 2500 California Plaza, Omaha, NE 68178, USA; ^2^Department of Pharmacy Practice, Creighton University, 2500 California Plaza, Omaha, NE 68178, USA

## Abstract

Chronic diseases like diabetes, hypertension, and dyslipidemia continue to be a significant burden on the US health care system. As a result, many healthcare providers are implementing strategies to prevent the incidence of heart disease and other chronic conditions. Among these strategies are proper drug therapy and lifestyle modifications. Behavior change is often the rate-limiting step in the prevention and maintenance of lifestyle modifications. The purpose of this paper is to describe a tool used to guide the progression and assess the effectiveness of a cardiovascular risk reduction program. The tool uses the Transtheoretical Model of Behavior Change to determine the readiness and confidence to change specific lifestyle behaviors pertinent to cardiovascular health. The tool aids the practitioner in developing a patient-centered plan to implement and maintain lifestyle changes and can be tailored to use in any situation requiring a behavior change on the part of the patient.

## 1. Introduction

Health behavior change is a key component in the prevention of disease. Studies have shown that 90% of type 2 diabetes, 80% of coronary artery disease, and 70% of all strokes are potentially preventable by a combination of nonsmoking, maintenance of a healthy bodyweight, regular physical activity, healthy eating habits, and moderate alcohol consumption [1]. Unfortunately, behavior change is often the rate-limiting step in the implementation and maintenance of these preventive lifestyle behaviors. 

The Transtheoretical Model of Behavior Change was developed to understand how individuals progress towards establishing and maintaining health behavior change for optimal health [2]. The model consists of six stages of change: precontemplation, contemplation, preparation, action, maintenance, and termination. The key to using this theory in practice is to assess the patient's stage and then educate and persuade the patient to move toward the action, maintenance, and termination stages [2]. The model is widely used by practitioners in the realm of substance abuse [3], as well as independently increasing physical activity [4] and proper nutrition [5]. However, there is little literature describing its practical use in a behavior change program that implements more than one behavior change.

In 2008, a comprehensive cardiovascular risk reduction program (CVRRP) was developed at a private Midwestern university to curb the progression of cardiovascular disease in its employees [6]. The program offers the participants an individualized lifestyle medication program that targets several behaviors including physical activity, nutrition, weight control, sleep success, stress reduction, alcohol intake, and tobacco cessation. A health behavior change tool was developed for the program to help identify which lifestyle behaviors to target first, set individual goals, and to assess both the program and its participants.

The purpose of this paper is to describe an innovative behavior change tool that is used to help modify health behaviors as part of a chronic disease risk reduction program and to present data obtained from that tool. The study was approved by the University's Institutional Review Board. 

## 2. Materials and Methods

### 2.1. Study Participants

The cardiovascular risk reduction program began as a pilot program at mid-sized private Midwestern university in 2008. The goal of the program is to reduce cardiovascular risk through education, medication management, and personalized lifestyle modification implementation. To be eligible for the program, the participant must be a university employee and carry the diagnosis of hypertension, dyslipidemia, diabetes, or a combination thereof. Each participant must meet one on one no fewer than twelve times with a clinical pharmacist. During the meetings, baseline risk assessment is completed, personalized lifestyle programs are developed, barriers to progress are addressed, and interventions are made as necessary. Several tools are used to improve awareness, education, adherence and communication in the program. These tools include a lifestyle journal, nutrition diary, pedometer, home blood pressure monitor, exercise facility incentive, a group newsletter, blog, and support group. A detailed description of the program was published previously [4]. 

The subjects described in this paper are a cohort of participants who began the program in 2009. There were thirty-five total participants: six males and twenty-nine females with an average age of 50.7 years when they started the program. Ten of the participants were diagnosed with diabetes, 22 with hypertension, and 20 with dyslipidemia.

### 2.2. Behavior Change Assessment and Tool Development

To assess readiness and confidence-to-change lifestyle behaviors, CVRRP participants completed two questionnaires at baseline and then again after six months of program participation (Appendices A and B). The “readiness-to-change” questionnaire incorporates the stages of the Transtheoretical Model in an easy-to-understand rating scale by which participants are asked to rank their readiness-to-change a variety of different lifestyle behaviors. The rating scale is listed at the top of the questionnaire and followed by a list of ten disease prevention activities. The disease prevention activities were chosen based on 2007 Health Risk Appraisal (HRA) data obtained from the university employees. According to the HRA data, these behaviors represented the significant areas in which changes were needed to improve employee health. At the time of this study's development, only five stages were described in the Transtheoretical Model. Since that time, a sixth stage, “termination”, was added. The questionnaires used in this study reflect only the first five stages of behavior change. 

Patients are first given the “readiness-to-change” questionnaire. The patient is asked to “indicate your readiness towards making changes/improvements in the lifestyle behaviors listed by using this five-point scale.” The Transtheoretical Model is then presented using the following scale:

(1) have been maintaining this lifestyle behavior for 6+ months,
(2) recently (within the previous 6 months) began this lifestyle behavior,
(3) plan to implement this lifestyle behavior sometime in the next month,
(4) thinking about implementing this lifestyle behavior sometime in the next few months,
(5) no interest in this lifestyle behavior at this time.

The behaviors assessed are related to exercise, nutrition, and living an overall healthy lifestyle. 

After completing the “readiness-to-change” questionnaire, the patient is given a second “confidence-to-change” questionnaire and asked to determine their level of confidence-to-change the same ten disease prevention activities. The rating scale offers three responses and is as follows:

(1) not very confident,
(2) somewhat Confident,
(3) very Confident.

### 2.3. Statistical Analysis

Questionnaire responses were analyzed using the Wilcoxon signed-rank test to see if there were significant differences of scores measured from baseline to 6 months. The Bonferroni correction for multiple comparisons was done as appropriate, and *P* values are described below. *P*  values < .005 were taken to indicate statistical significance.

## 3. Results

The baseline and six-month ratings for the “readiness-to-change” questionnaire are shown in Table 1. The ratings are listed as aggregate mean values for the subjects under evaluation. Categories with a statistically significant increased change in readiness (*P* < .005) were incorporating extra physical activity throughout the daily routine, implementing specific lifestyle strategies to help with lifestyle modifications, and living an overall healthy lifestyle. These three categories also showed the greatest degree of difference from baseline to six months. 

The lowest rating at both baseline and six months came from avoiding smoking or tobacco use. The data indicated that participants have been maintaining this lifestyle behavior for six months or longer. Of note, there was only one smoker in the cohort at baseline. At baseline, the participants were least interested in starting a sporting activity one to two or more times per week. At six months, this value was one of two that shifted upward (toward a rating of less interest in changing). The other category in which readiness decreased was that of smoking and tobacco cessation/avoidance. Each of the other categories showed a positive change toward increased readiness-to-change, plans to implement, and beginning or maintaining healthy lifestyle behaviors. By six months, participants rated each of the lifestyle behaviors listed on the survey between 1.14 and 2.19 (with the exception of participating in a sporting activity). These values indicate that participants had recently implemented or have been maintaining each of the behaviors into their lifestyle.

The mean baseline and six-month ratings for the confidence-to-change questionnaire are shown in Table 2. There were no statistically significant changes in patient's confidence-to-change behaviors. Participants were most confident about avoiding smoking and tobacco use. There was only one smoker enrolled in the program. 

The category ranked the second highest in confidence-to-change was the ability to implement specific lifestyle strategies to help with lifestyle modifications. The categories with the highest degree of increased confidence were incorporating purposeful exercise five times per week or more (0.17), incorporating extra physical activity throughout the day (0.15), and eating five or more fruits and vegetables each day (0.15). The least confident area of lifestyle behavior change was participating in a sporting activity at least one to two times per week (1.35). There were five categories were participant's confidence decreased over the first six months of the program. Confidence to lose or maintain body weight decreased the most (−0.16), followed by confidence to avoid smoking and tobacco use (−0.09) and confidence to implement strategies to help with lifestyle modifications (−0.08). There was a slight decrease in confidence to consume foods high in fiber (−0.03) and to choose whole-grain foods (−0.02). Across the spectrum of lifestyle behaviors surveyed (0.68 − 0.38 = 0.30), confidence to positively change lifestyle was increased.

## 4. Discussion

As a standard of practice, the data from the completed questionnaires are used by the clinical staff to determine which behaviors to address first, help set patient-driven goals, determine what the patient is not interested in changing, and determine the participant's stage of readiness to consistently participate in a behavior. The information from the questionnaires can also be used to assess the efficacy and progress of the program itself, as well as the progression of the patient.

The primary purpose of administering the questionnaires at the baseline visit is to identify which preventive activities to implement first and set goals to drive the next meeting. Categories that participants have recently implemented or have been maintaining (ratings of 1 or 2 on the “readiness-to-change” survey) should be commended and can be used as positive reinforcement throughout the course of the program. If patients rate their confidence to continue to these activities as “somewhat” or “not very” confident, a discussion can take place regarding the barriers to maintaining the lifestyle and strategies to continue the behavior. 

Next, the activities that are ranked a “3” or “4” are discussed. These are the behaviors the patient is thinking or planning to implement and should drive the goal-setting process for the patient. The confidence scores are again used to determine which behavior to target first. The activities that are rated with high confidence generally require less work to adopt into daily routine. Achieving initial success can increase patient confidence to tackle additional activities in the future. Behaviors with lower confidence ratings present great opportunities to educate, encourage and address patient-specific barriers to implementation, and develop strategies to overcome the barriers.

Finally, activities rated with a “1” or “no interest in this lifestyle behavior at this time” are discussed. These behaviors are not targeted first, and the discussion revolves around why there is no interest in changing the lifestyle. These activities can be readdressed at a later time.

The primary purpose of the follow-up questionnaires is to help guide the ongoing lifestyle medicine program, modify patient specific goals, and assess patient's individual progress towards achieving their goals. 

The program and goals are always set based on individual responses; however, the aggregate readiness and confidence data showed a similar trend at baseline and six months. The goals of “implementing lifestyle strategies to help with lifestyle modifications” and “live an overall healthy lifestyle” were removed due to nonspecificity. Additionally, the goal “avoid smoking or tobacco use” was removed because only one person smoked, and the goal “participate in a sporting activity at least 1-2 times/week” was removed because of little interest. The remaining goals were ranked based on mean participant responses. The activity ranked at “1” was the behavior participants were most ready to change and most confident to change. As the ranking number increased, the participants' readiness and confidence-to-change the behavior decreased. After the above-stated behaviors were removed, the remaining six behaviors were ranked in very similar orders at baseline and six months. The behaviors that ranked from one to four were consistently choose foods with whole grains, incorporate extra physical activity, choose foods high in fiber, and eat five or more servings of fruits and vegetables daily. The two lifestyle behaviors that consistently ranked five and six were exercise five or more times weekly and lose or maintain bodyweight. This data interestingly reflects that as a whole, participants are more ready and confident to make dietary changes in their lifestyle than physical activity and weight changes.

The results of the present study indicate that a CVRRP can increase the readiness and confidence-to-change specific lifestyle behaviors that relate to overall cardiovascular health. The ultimate goal of the program is to prompt patients to start thinking about healthy lifestyles and implementing them into their daily routine. The six-month data from the readiness-to-change portion of this study indicate that this goal is being met. With the exception of participating in a sporting activity, each of the other categories ranked below 2.19, which suggests that participants had recently (within the last six months) implemented the modification or that they had been maintaining the behavior for twelve or more months. 

A nutrition study performed by Van Duyn and associates [7] suggested that survey responses from questionnaires incorporating the Transtheoretical Model can successfully be used to predict actual fruit and vegetable consumption in human subjects. Although the data from the current study is patient reported and does not include actual lifestyle data from the participants, it can still successfully be used to predict actual behavior. 

The authors hypothesized that confidence would increase after participation in the program. After six months, five of the ten categories actually showed a decrease in confidence-to-change lifestyle behavior. This decreased confidence may be due to the increased awareness of the time and work required to maintain the lifestyle behavior. Because there was only one smoker in the cohort assessed, additional studies with larger smoking populations are necessary to interpret the usefulness of the tool in changing the behavior of tobacco use.

The activities assessed in the current study are not all-inclusive for lifestyle medicine or disease prevention. Because sleep [8], stress [9], and alcohol intake [10] are associated with cardiovascular health and overall quality of life, additional questions could be added to the current questionnaires to assess these lifestyle behaviors within the program. This tool can be easily tailored to work wherever the Transtheoretical Model is used in practice. The tool can be used in a variety of practice settings including physician run clinics, pharmacies, and dieticians' offices. With minor adjustments, the tool can be used for weight loss programs or chronic disease management programs with a specific focus on diabetes, asthma, or dyslipidemia.

## 5. Conclusions

Health behavior change is often considered the rate-limiting step in lifestyle medicine programs. The “readiness-to-change” and “confidence-to-change” tools can effectively be used to implement health behavior change strategies at a patient-specific level. The tool can be tailored to fit any program aiming to change behavior and can be applied to a variety of practice types.

## Figures and Tables

**Table 1:** Readiness-to-change lifestyle data.

Readiness-to-change data				
	Baseline rating	6-month rating	Change in ratings	*P* value
Purposeful exercise at least 5x/week	2.85	2.07	−0.78	.013
Incorporate “extra” physical activity through the daily routine	2.56	1.71	−0.85	.001
Participate in a sporting activity at least 1-2 times/week	4.24	4.29	0.05	.448
Eat 5+ servings of fruits and vegetables daily	2.58	2.00	−0.58	.015
Consistently choose foods with whole grains	2.12	1.96	−0.16	.258
Choose foods high in fiber	2.36	2.04	−0.32	.070
Lose or maintain body weight	2.58	2.19	−0.39	.319
Avoid smoking or tobacco use	1.12	1.14	0.02	.180
Implement specific lifestyle strategies to help with lifestyle modifications	2.85	1.96	−0.90	.003
Live an overall health lifestyle	2.70	2.00	−0.70	.004

**Table 2:** Confidence-to-change lifestyle data.

Confidence-to-change data				
	Baseline rating	6-month rating	Change in ratings	*P* value
Purposeful Exercise at least 5x/week	2.26	2.43	0.17	.617
Incorporate “extra” physical activity through the daily routine	2.56	2.71	0.15	.257
Participate in a sporting activity at least 1-2 times/week	1.35	1.46	0.11	.429
Eat 5+ Servings of fruits and vegetables daily	2.53	2.68	0.15	.166
Consistently choose foods with whole grains	2.56	2.54	−0.02	.644
Choose foods high in fiber	2.53	2.50	−0.03	1.000
Lose or maintain body weight	2.41	2.25	−0.16	.124
Avoid smoking or tobacco use	2.91	2.82	−0.09	.414
Implement specific lifestyle strategies to help with lifestyle modifications	2.62	2.54	−0.08	.617
Live an overall health lifestyle	2.47	2.57	0.10	.527

## Appendices

### A. Readiness-to-Change Lifestyle Questionnaire

Name:___________________

Date: ___________________

Cardiovascular Risk Reduction Program Readiness-to-Change Lifestyle Behavior

Indicate the readiness of the patient towards making changes/improvements in the lifestyle behaviors listed below by using this 5-point scale.

**Table:**

Rating	Readiness-to-change

5	No interest in this lifestyle behavior at this time
4	Thinking about implementing this lifestyle behavior sometime in the next few months
3	Plan to implement this lifestyle behavior sometime in the next month
2	Recently (within the previous 6 months) began this lifestyle behavior
1	Have been maintaining this lifestyle behavior for 6+ months

Rating	Lifestyle behavior

—	Purposeful exercise at least 5 times per week
—	Incorporate “extra” physical activity throughout the daily routine (“extra” = taking stairs rather than elevator, walking pets, parking further away in parking lot, etc.)
—	Participate in sporting activity at least 1-2 times weekly (e.g., golf, volleyball, basketball, tennis)
—	Eat 5 or more servings of fruits and vegetables daily
—	Consistently choose foods with whole grains
—	Choose foods high in fiber
—	Lose or maintain body weight
—	Avoid smoking or tobacco use
—	Implementing specific strategies to help with lifestyle modifications
—	Live an overall healthy lifestyle.

### B. Confidence-to-Change Lifestyle Questionnaire

Name:_______________________

Date: _______________________

Cardiovascular Risk Reduction Program Confidence-to-Change Lifestyle Behavior

Rate the following statements based on the 3-point scale below.

**Table:**

Rating	Confidence-to-change

3	Very confident
2	Somewhat confident
1	Not very confident

Rating	Lifestyle Behavior

—	Purposeful exercise at least 5 times per week
—	Incorporate “extra” physical activity throughout the daily routine (“extra” = taking stairs rather than elevator, walking pets, parking further away in parking lot, etc.)
—	Participate in sporting activity at least 1-2 times weekly (e.g., golf, volleyball, basketball, tennis)
—	Eat 5 or more servings of fruits and vegetables daily
—	Consistently choose foods with whole grains
—	Choose foods high in fiber
—	Lose or maintain body weight
—	Avoid smoking or tobacco use
—	Implementing specific strategies to help with lifestyle modifications
—	Live an overall healthy lifestyle
	
